# Single-strain probiotics enhance growth, anti-pathogen immunity, and resistance to *Nocardia seriolae* in grey mullet (*Mugil cephalus*) via gut microbiota modulation

**DOI:** 10.1186/s42523-024-00353-0

**Published:** 2024-11-19

**Authors:** Ching-Hung Chan, Li-Han Chen, Kuang-Yu Chen, I-Hung Chen, Kung-Ta Lee, Liang-Chuan Lai, Mong-Hsun Tsai, Eric Y. Chuang, Ming-Tse Lin, Tsong-Rong Yan

**Affiliations:** 1 Department of Chemical Engineering and Biotechnology, College of Engineering, Tatung University, Taipei, Taiwan; 2 Institute of Fisheries Science, College of Life Science, National Taiwan University, Taipei, Taiwan; 3 Department of Life Science, College of Life Science, National Taiwan University, No. 1, Sec. 4, Roosevelt Rd., Taipei, 106319 Taiwan; 4 Department of Biochemical Science & Technology, College of Life Science, National Taiwan University, Taipei, 10617 Taiwan; 5 Graduate Institute of Physiology, College of Medicine, National Taiwan University, Taipei, Taiwan; 6 Bioinformatics and Biostatistics Core, Center of Genomic and Precision Medicine, National Taiwan University, Taipei, Taiwan; 7 Institute of Biotechnology, National Taiwan University, Taipei, Taiwan; 8 Graduate Institute of Biomedical Electronics and Bioinformatics, National Taiwan University, Taipei, Taiwan; 9 Biomedical Technology and Device Research Laboratories, Industrial Technology Research Institute, Hsinchu, Taiwan

**Keywords:** Probiotics, Gut microbiota, Disease resistance, *Mugil cephalus*, *Nocardia seriolae*

## Abstract

Grey mullet (*Mugil cephalus*) aquaculture is economically vital due to the high value of its roe. However, it faces significant risks from disease outbreaks, particularly from *Nocardia seriolae*. Current reliance on antibiotics has drawbacks, highlighting the potential of probiotics as a promising alternative. Despite this, no studies have focused on the effects and mechanisms of probiotics in disease prevention and treatment in grey mullet. This study, therefore, investigates the efficacy of probiotics in enhancing disease resistance and promoting growth in grey mullet. Three strains of probiotics, *Lacticaseibacillus rhamnosus *FS3051, *Limosilactobacillus reuteri *FS3052, and *Bacillus subtilis* natto NTU-18, were selected to evaluate their anti-*N. seriolae* activity and hydrolytic enzyme secretion in vitro. Then, 144 grey mullet were randomly divided into four groups: control, *L. rhamnosus *FS3051, *L. reuteri *FS3052, and *B. subtilis* natto NTU-18. After being fed the corresponding diet for 28 days, fish were measured for immune gene expression and short-term growth followed by challenge of *N. seriolae*. Survival rates were recorded for 35 days post challenge. Additionally, the gut microbiota of the control and probiotic groups with effects on both growth and protection against *N. seriolae* were analyzed to investigate the potential role of gut microbiota. Results demonstrated that *L. rhamnosus* FS3051 and *L. reuteri* FS3052 inhibited *N. seriolae*, while *B. subtilis* natto NTU-18 did not inhibited *N. seriolae*. Probiotics also had the ability to secrete hydrolytic enzymes. Probiotic-fed grey mullet showed significant improvements in weight gain ratio, feed efficiency, and specific growth rate, particularly in the *B. subtilis* natto NTU-18 group. Immune gene expression was enhanced by probiotics, especially *L. rhamnosus*, FS3051, which induced IL-8, IL-1β, TNF-α, IFN-γ, and MHCI. Survival rates post-*N. seriolae* challenge improved significantly for *L. rhamnosus *FS3051-fed fish. *L. rhamnosus* FS3051 also altered the gut microbiota, enriching beneficial genera like *Lactobacillus*, which correlated positively with immune responses and growth, while reducing *Mycoplasma* and *Rhodobacter*, which were negatively correlated with immune responses. This study underscores the potential of probiotics in enhancing disease resistance and growth via regulating gut microbiota in grey mullet.

**Supplementary Information:**

The online version contains supplementary material available at 10.1186/s42523-024-00353-0.

## Introduction

Grey mullet (*Mugil cephalus*) aquaculture in East Asia is of significant economic importance, primarily due to the high value associated with its roe, which is sought after as a premium festive gift in many countries, thereby contributing substantially to aquaculture production value [1]. However, the cultivation of grey mullet presents considerable risks owing to its lengthy cultivation period of over two years necessary for optimal roe yields. Compounded by the low market price of its meat, grey mullet aquaculture typically allows for only one harvest opportunity annually. Given these factors, mitigating the risks associated with grey mullet aquaculture is imperative.

Disease outbreaks pose a significant threat to aquaculture, particularly for species like grey mullet with extended cultivation periods and limited harvesting seasons. Thus, reducing disease incidence is crucial and can yield substantial benefits. Aquatic disease outbreaks are multifaceted phenomena involving environmental factors, pathogens, and cultured species, necessitating comprehensive research to develop effective mitigation strategies. Current management practices often rely on antibiotics to control bacterial diseases in aquaculture, but such approaches pose health risks to consumers and contribute to environmental pollution [2]. For example, *Nocardia seriolae* is one of the major pathogens in grey mullet [3] and several fish species [4–6] and causes up to 60% mortality in grey mullet [7]. Currently, the application of antibiotics was the most efficient strategy to prevent *N. seriolae* in aquaculture. To address these challenges derived by antibiotics, probiotics have emerged as promising tools in recent years, with research indicating their potential to enhance disease resistance in fish [8].

Probiotics play multifaceted roles in fish nutrition, disease resistance, and various beneficial activities, with immune system modulation being prominently cited [9]. These microorganisms often exhibit antagonistic activities, secrete extracellular enzymes, compete for colonization sites, and regulate immunity, rendering them widely applicable across diverse animal species. Moreover, probiotics can influence the host’s comprehensive physiological function via regulating gut microbiota [10]. The rapid growth of the aquaculture industry has spurred extensive research into incorporating probiotics into aquafeeds to bolster disease resistance and feed efficiency [9]. Among the common probiotics in aquaculture, *Lactobacillus* and *Bacillus* stand out. For instance, numerous probiotic strains, such as *Lactobacillus acidophilus* [11], *Lactobacillus reuteri* (*Limosilactobacillus reuteri*) [12], and *Lactobacillus plantarum* (*Lactiplantibacillus plantarum*) [13], have been documented to upregulate the expression of various cytokines in host organisms, indicating their immunomodulatory effects. In rainbow trout, a mixture of different strains of *Lactobacillus rhamnosus* (*Lacticaseibacillus rhamnosus*) led to significant enhancements in serum lysozyme, complement activity, and head kidney leukocyte phagocytic activity [14]. *Bacillus* species, known for their survival advantages in stressful environments, produce metabolites with antagonistic effects against pathogenic microorganisms and possess immune-regulating capabilities. For instance, supplementation with *Bacillus subtilis* C-3102 in tilapia feed enhances mucosal adhesion and elevates the expression of intestinal cytokines such as IL-1β, TGF-β, and TNF-α, contributing to improved immune responses [15]. Pillinger et al. (2022) also suggested that *B. subtilis* employed the mechanism that enhanced the immune responses to prevent rainbow trout against pathogens [16]. Additionally, *Bacillus* promotes feed utilization and fish growth rate, potentially through bacterial synthesis of digestive enzymes or stimulation of endogenous enzyme production in tilapia [15]. Overall, *Lactobacillus* and *Bacillus* hold significant promise for diverse applications in aquatic probiotics. However, to our knowledge, no study has demonstrated the efficacy of probiotics in enhancing the resistance of grey mullet against *N. seriolae*.

In this study, we aimed to select probiotics that could enhance growth performance and resistance against one of the most serious pathogens, *N. seriolae*, in grey mullet. We focused on *L. rhamnosus *FS3051, *L. reuteri *FS3052, and *B. subtilis* natto NTU-18, considering their benefits to various fish species in previous studies [9, 12, 17]. Our objectives included investigating the anti-*N. seriolae* capabilities and hydrolytic enzyme secretion of these probiotics in vitro, evaluating their effects and mechanism on growth performance, immunity, and gut microbiota of grey mullet after 28 days of feeding, and assessing their protective effects against *N. seriolae* by the challenge trial following a 28-day probiotic feeding period.

## Materials and methods

### Ethical considerations

All animal experiments were conducted in accordance with protocols approved by the Institutional Animal Care and Use Committee (IACUC) of National Taiwan University (NTU-109-EL-00153).

### Pathogen and probiotics

*N. seriolae* (provided by Professor Mei-Mei Chen, National Taiwan University) was cultured in Tryptone Soy Broth (STBIO, New Taipei, Taiwan) at 28 °C for 5 days. *L. rhamnosus* FS3051 and *L. reuteri* FS3052 were isolated from local pickles and cultured in MRS medium (STBIO) for 24 h. *B. subtilis* natto NTU-18 (BCRC 80390, Bioresource Collection and Research Center, Taiwan) was isolated from fermented natto and cultured in Luria-Bertani broth (LB broth) (STBIO) for 24 h.

### Inhibition assay against N. seriolae in vitro

The antimicrobial activity of probiotics against *N. seriolae* was evaluated using the disk diffusion method, as adapted from prior protocols [18]. Specifically, paper disks (8 mm in diameter) saturated with either *L. rhamnosus *FS3051, *L. reuteri *FS3052, or *B. subtilis* natto NTU-18, with 60 µl supernatant of ×10^8^ CFU/mL were prepared and positioned onto Tryptic Soy Broth (TSB) agar plates (Neogen, Lansing, MI, USA) where *N. seriolae* were previously evenly spread with culture medium. The plates were then incubated for 24 h at 30 °C. Subsequently, the efficacy of probiotics against *N. seriolae* was determined by measuring the diameter of the bacteriostatic zone observed around the paper disks on the TSB agar plates.

### In vitro determination of enzyme activity

The enzyme activity assays were adapted from previous studies [19, 20] with some modifications. For all assays, 10 µl of either probiotic (10^5^ CFU/ml) or culture medium were added onto 8 mm paper disks placed on the agar containing the enzyme-specific substrate. Then, the plates were incubated at 37 °C for 24 h. For the starch hydrolysis assay, starch medium agar was used, consisting of beef extract (5 g/L), peptone (10 g/L), NaCl (5 g/L), soluble starch (2 g/L), and agar (20 g/L) (STBIO). After incubation, the plates were staint with Lugol’s iodine solution (HiMedia, Mumbai, India). For the proteinase and lipase hydrolysis assays, skim milk agar and tributyrin agar were used, respectively. Skim milk agar was composed of skim milk powder (28.0 g/L), tryptone (5.0 g/L), yeast extract (2.50 g/L), dextrose glucose (1.0 g/L), and agar (15.0 g/L) (STBIO), while tributyrin agar consisted of peptone (5 g/L), yeast extract (3 g/L), and agar (15 g/L) (STBIO). For the cellulase hydrolysis assay, cellulose agar was used, containing K_2_HPO_4_ (1 g/L), MgSO_4_·7H_2_O (0.5 g/L), KCl (1 g/L), CMC-Na salt (5 g/L), yeast extract (0.5 g/L), NaNO_3_ (1 g/L), glucose (1 g/L), and agar (17.5 g/L) (STBIO). After incubation, the cellulose agar plates were stained with 0.02% Congo red solution (Sigma-Aldrich, St. Louis, MO, USA) for 20 min, then washed twice with 0.9% saline solution for 10 min each. Enzyme activity was indicated by the appearance of clear zones around the paper disks, which were measured for analysis.

### Experimental diet

The probiotics were initially centrifuged at 8000 *g*, and the settled pellets were then resuspended in PBS and sprayed on commercial diet (local supplier) at a concentration of 10^9^ CFU/g. The control diet was mixed with PBS only. The diet was dried in the laminar flow at room temperature for 30 min and stored at 4 °C until use.

### Experimental grey mullet

A total of 144 grey mullet (body weight (mean): 1.07 ± 0.07 g; body length (mean): 43.4 ± 1.47 mm) were purchase from a local aquaculture farm (Tainan, Taiwan) and housed under standard laboratory conditions, with a 12/12-hour light/dark cycle and a temperature of 24–26 °C. After one week adaptation period, fish were randomly divided into four groups, each comprising 36 individuals in 4 tanks (3 tanks with 10 fish for challenge trial, 1 tank with 6 fish for trials of growth, gene expression, and gut microbiota). The fish culture system is following the description in previous study [21]. Fish were reared in an indoor recirculating aquaculture system with a capacity of 30 L/tank. Throughout the rearing period, the tanks were supplied with UV-treated and filtered water, and 50% of the water was replaced twice a week. These groups were given either regular (control) or one of the probiotic diets (experimental groups) twice a day for 28 days. The duration of probiotic treatment was referred to the previous study [22] and out pilot study. The weight and length of fish were measured at the 0 day and 28 days and calculated as follows:

$$\:\text{W}\text{e}\text{i}\text{g}\text{h}\text{t}\:\text{g}\text{a}\text{i}\text{n}\:\text{r}\text{a}\text{t}\text{e}\:(\text{W}\text{G}\text{R},\:\text{\%})\:=\frac{(\text{W}\text{f}\hspace{0.17em}-\hspace{0.17em}\text{W}\text{i})}{\text{W}\text{i}\times\:100}$$

$$\:\text{L}\text{e}\text{n}\text{g}\text{t}\text{h}\:\text{g}\text{a}\text{i}\text{n}\:\text{r}\text{a}\text{t}\text{e}\:(\text{L}\text{G}\text{R},\:\text{\%})\:=\frac{(\text{L}\text{f}\hspace{0.17em}-\hspace{0.17em}\text{L}\text{i})}{\text{L}\text{i}\times\:100}$$

$$\:\text{F}\text{e}\text{e}\text{d}\:\text{e}\text{f}\text{f}\text{i}\text{c}\text{i}\text{e}\text{n}\text{c}\text{y}\:\left(\text{F}\text{E}\right)\:=\frac{(\text{W}\text{f}\hspace{0.17em}-\hspace{0.17em}\text{W}\text{i})}{\text{t}\text{o}\text{t}\text{a}\text{l}\:\text{f}\text{e}\text{e}\text{d}\:\text{i}\text{n}\text{t}\text{a}\text{k}\text{e}}$$

$$\:\text{S}\text{p}\text{e}\text{c}\text{i}\text{f}\text{i}\text{c}\:\text{g}\text{r}\text{o}\text{w}\text{t}\text{h}\:\text{r}\text{a}\text{t}\text{e}\:(\text{S}\text{G}\text{R},\:\%\:\text{d}^{-1})=100\times\frac{(\text{l}\text{n}\:\text{W}\text{f}-\text{l}\text{n}\:\text{W}\text{i})}{\text{d}\text{a}\text{y}\text{s}}$$

(Wf: final weight; Wi: initial weight; Lf: final length; Li: initial length)

### RNA isolation and RT-qPCR

Spleen and liver of grey mullet was collected at the 28th day. RNA isolation was carried out using the RNeasy Mini kit (Qiagen, Hilden, Germany). Subsequently, 500 ng of RNA from each sample underwent reverse transcription using the iScript cDNA Synthesis Kit (Bio-Rad, USA), following the manufacturer’s instructions. qPCR analysis was conducted using the MyiQ Single-Color Real-Time PCR Detection System (Bio-Rad, Hercules, CA, USA). Gene-specific primers (Purigo, Taipei, Taiwan) utilized in the study were referred to the previous study [23] and listed in Table 1. β-actin served as an internal control to normalize the mRNA levels of the tested genes.

**Table 1:** Primer sequences

Gene		Sequences
IL-1β	Forward	GAGGAGCTTGGTGCAGAACA
	Reverse	CTTTGTTCGTCACCTCCTCCA
IL-8	Forward	CACTGCTGGTCGTCCTCATT
	Reverse	CAGTCGGAGGTCGGAAGTCT
TNF-α	Forward	GCGCAGTCTGTCATTGGTT
	Reverse	ACTGGACACGCTCACTGTAGTG
TLR2	Forward	GGCTCCAGCAGGATCAAAAT
	Reverse	CTTCCTACCAGGTCACTGGAT
IFN-γ	Forward	GAGGGAAAGTGTGACCAAAGA
	Reverse	GGCTGCTGGTCCATTACAATA
C3	Forward	GCATCACGCTCCTTGTCTTT
	Reverse	ACCACTATGCCACAAGAACATC
MHCI	Forward	GCAGAACCAGAGGCTTCAACA
	Reverse	TCAGGAGGAGTTGTGTCTATGAAC
β-actin	Forward	TGCAGTCAACATCTGGAATC
	Reverse	ATTTTTGGCGCTTGACTCAG

### Challenge test

The challenge test followed the previous study with slight modifications [24]. Briefly, the *N. seriolae* were introduced into the tank containing 10 fish and 1 L of water, resulting in a *N. seriolae* concentration of 5 × 10^5^ CFU/ml. After 1 hour immersion, the fish each tank were transferred to a 30 L freshwater tank. Daily recordings of fish mortality rates were conducted, and observations of fish behavior and condition were made for 35 days post infection. To confirm the infection of *N.seriolae*, the DNA was isolated from the liver of dead fish using QIAamp DNAkit (Qiagen). Then, *N. seriolae* were detected using PCR with specific primers NS1: 5’-ACTCACAGCTCAACTGTGG-3’ and NG1:5’-CCGACCACAAGGGGG-3’ following the description in Miyoshi and Suzuki’s study [25].

### Gut microbiome analysis

Gut microbiome analysis was performed in the control fish and fish fed probiotics with effects on both growth and protection against *N. Seriolae* (n = 3). The DNA was isolated from the gut at the 28th day using QIAamp DNA Microbiome Kit (Qiagen) according to the manufacturer’s instructions. The 16S rRNA gene sequencing and subsequent data analysis procedures closely followed those outlined in the previous study [26]. Briefly, the DNA quality and quantity and PCR amplification of the V3–V4 region of the 16S rRNA gene was conducted using primers 341 F (5’-CCTAYGGGRBGCASCAG-3’) and 806 R (5’-GGACTACNNGGGTATCTAAT-3’), as per the Illumina 16 S Metagenomic Sequencing Library Preparation manual. Amplicon libraries were generated according to Illumina’s protocol and subjected to paired-end sequencing (2 × 250) on an Illumina HiSeq 2000 platform. Following quality control, forward and reverse reads were merged, and operational taxonomic units (OTUs) were constructed at 97% identity using the UPARSE pipeline (drive5, Tiburon, California), with mapping to the SLIVA database v.138 [27]. Subsequently, data analysis was conducted using Quantitative Insights Into Microbial Ecology (QIIME2) [28], with removal of chimeric sequences employing ChimeraSlayer. Sequences sharing ≥ 97% similarity were grouped into the same OTU. Alpha diversity analysis, utilizing Shannon, was performed with QIIME2, while beta diversity was assessed via principal coordinate analysis. Statistical significance of beta diversity was determined using PERMANOVA. Additionally, linear discriminant analysis effect size (LEfSe) was executed online within the Galaxy workflow framework.

### Statistical analyses

Data are expressed as the mean ± standard error. One-way ANOVA with Tukey’s HSD posttest was used to analyze the difference between the groups after the homogeneity of variance was tested. The survival probability of fish post challenge was analyzed using the Kaplan–Meier method and log-rank tests were used to compare survival curves. Spearman’s correlation coefficient was employed to assess the relationship between bacterial abundance and physiological parameters. Statistical significance was set at a *p* value < 0.05.

## Results

### Evaluation of probiotics capabilities in vitro

The capability of probiotics, *L. rhamnosus *FS3051, *L. reuteri *FS3052 and *B. subtilis* natto NTU-18, were investigated in vitro. The supernatants of these probiotics were used to test the inhibition of *N. seriolae*. The diameters of the inhibition zones were shown in the Table 2. Additionally, the activity of hydrolytic enzymes secreted by the probiotics was assessed. *L. rhamnosus* FS3051 and *B. subtilis* natto NTU-18 exhibited protease, amylase, and cellulase activity, while *L. reuteri* FS3052 showed protease activity. None of the probiotics exhibited lipase activity (Table 3).

**Table 2:** Ability of probiotics against *Nocardia seriolae*

	*Lacticaseibacillus rhamnosus* FS3051	*Limosilactobacillus reuteri* FS3052	*Bacillus subtilis* natto NTU-18
Diameter of inhibition zone (mm)	11.94 ± 0.72	11.38 ± 0.88	-

-: without clear zone

**Table 3:** Ability of probiotics in secretion of digestive enzymes

	Protease	Amylase	Lipase	Cellulase
*L. rhamnosus FS3051*	20.15 ± 0.18	13.88 ± 0.26	-	11.97 ± 0.6
*L. reuteri FS3052*	18.49 ± 0.01	-	-	-
*B. subtilis* natto NTU-18	27.97 ± 0.27	17.23 ± 0.27	-	10.92 ± 0.52

-: without clear zone

$: Diameter of inhibition zone (mm)

### The probiotics promoted the growth of grey mullet

To assess the effect of the probiotics on growth performance of grey mullet, the WGR, LGR, FE, and SGR were evaluated after feeding the probiotics for 28 days. Initial body weight and length showed no significant differences. Supplementation with *L. rhamnosus *FS3051,* L. reuteri *FS3052, and *B. subtilis* natto NTU-18 resulted in more than a 5% increase in body length gain compared to the control group. Moreover, WGR, LGR, FE, and SGR were significantly higher in the groups fed *L. rhamnosus* FS3051 and *B. subtilis* natto NTU-18 compared to the control group. Specifically, the group fed *B. subtilis* natto NTU-18 exhibited the highest values for WGR, LGR, FE, and SGR at 58.63 ± 19.08%, 14.49 ± 2.67%, 69.80 ± 22.71%, and 1.63 ± 0.42, respectively (Table 4). Raw body weight and length data are in the supplementary material.

**Table 4:** Effect of probiotics on growth of grey mullet in 28 days*

	Control	*Lacticaseibacillus rhamnosus* FS3051	*Limosilactobacillus reuteri* FS3052	*Bacillus subtilis* natto NTU-18
Wi (g)	1.03 ± 0.08^a^	1.09 ± 0.04^a^	1.08 ± 0.06^a^	1.09 ± 0.06^a^
Wf (g)	1.30 ± 0.10^a^	1.66 ± 0.15^b^	1.67 ± 0.05^b^	1.72 ± 0.15^b^
Li (mm)	42.78 ± 2.33^a^	42.65 ± 0.04^a^	43.77 ± 1.49^a^	43.40 ± 0.59^a^
Lf (mm)	45.53 ± 1.84^a^	48.33 ± 2.02^ab^	49.01 ± 2.00^b^	49.69 ± 1.04^b^
WGR(%)	25.99 ± 2.31^a^	51.62 ± 7.94^b^	54.29 ± 12.81^b^	58.63 ± 19.08^b^
LGR(%)	6.48 ± 2.04^a^	13.33 ± 3.50^b^	11.97 ± 1.35^b^	14.49 ± 2.67^b^
FE(%)	30.94 ± 2.75^a^	61.46 ± 9.46^b^	64.63 ± 15.25^b^	69.80 ± 22.71^b^
SGR(%)	0.82 ± 0.07^a^	1.48 ± 0.18^b^	1.54 ± 0.30^b^	1.63 ± 0.42^b^

Different superscript letters (a, b) differ significantly at *p* < 0.05 by one-way ANOVA with Tukey’s HSD posttest. *n* = 6. Wi: initial body weight; Wf: final body weight; Li: initial body length; Lf: final body length; WGR: body weight gain rate; LGR: body length gain rate; FE: feed efficiency; SGR: specific growth rate

### Immune responses were enhanced by the probiotics

**Fig. 1:**
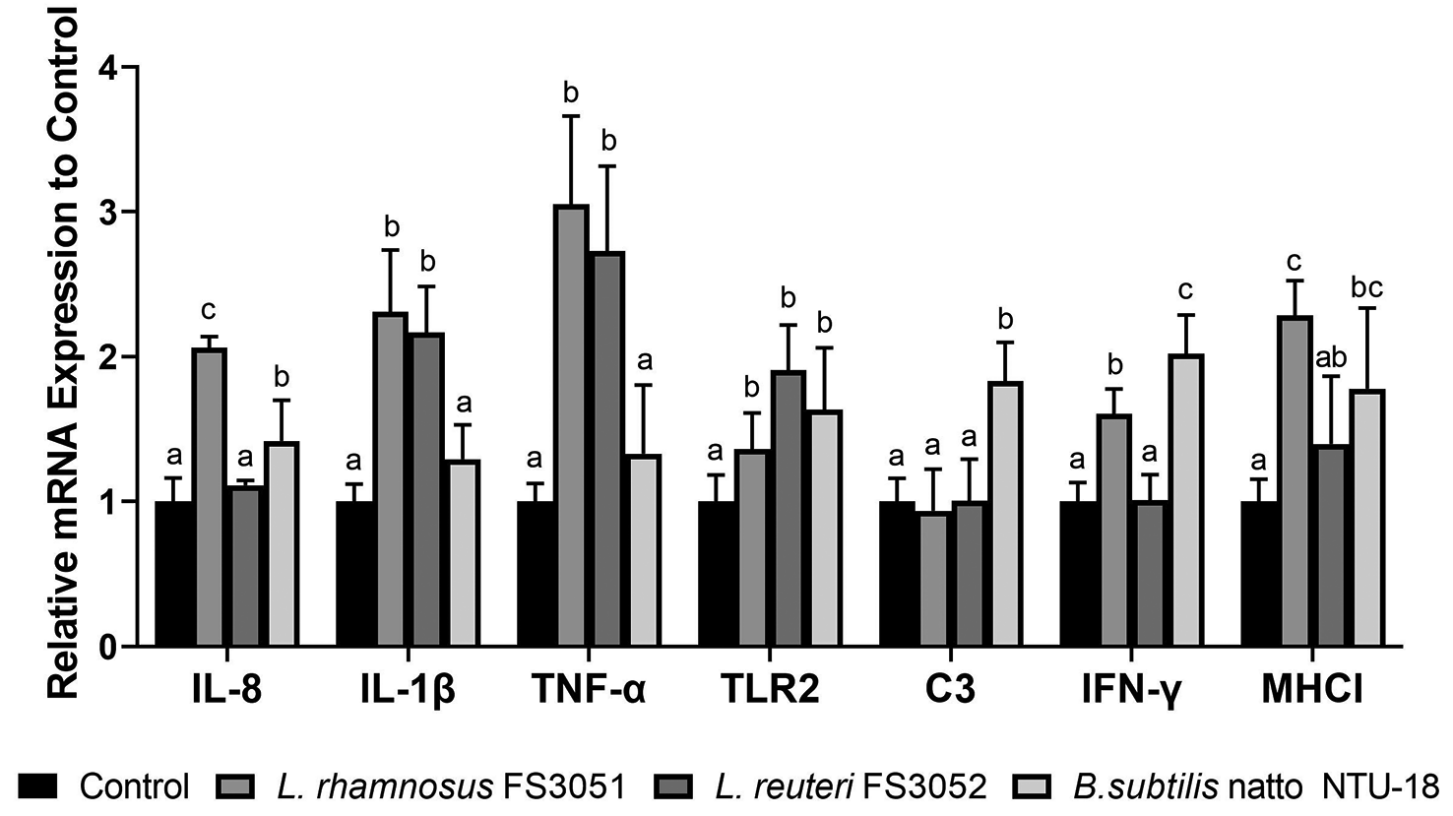
Expression of immune genes after 28-day probiotic supplementation. Different superscript letters (a, b, c) differ significantly at *p* < 0.05 by one-way ANOVA with Tukey’s HSD posttest. *n* = 6

Immune responses of grey mullet were evaluated after 28 days of probiotic feeding. *L. rhamnosus* FS3051 induced IL-8, IL-1β, TNF-α, IFN-γ, and MHCI, showing the strongest effects on IL-8, IL-1β, TNF-α, and MHCI among the groups. *B. subtilis* natto NTU-18 increased the expression of immune genes, including IL-8, MHCI, TLR2, IFN-γ, and C3. Additionally, IL-1β, TNF-α, and TLR2 were induced by *L. reuteri* FS3052 (Fig. 1).

### Supplement of *L. rhamnosus* FS3051 increased the survival of grey mullet post *N. seriolae* challenge

**Fig. 2:**
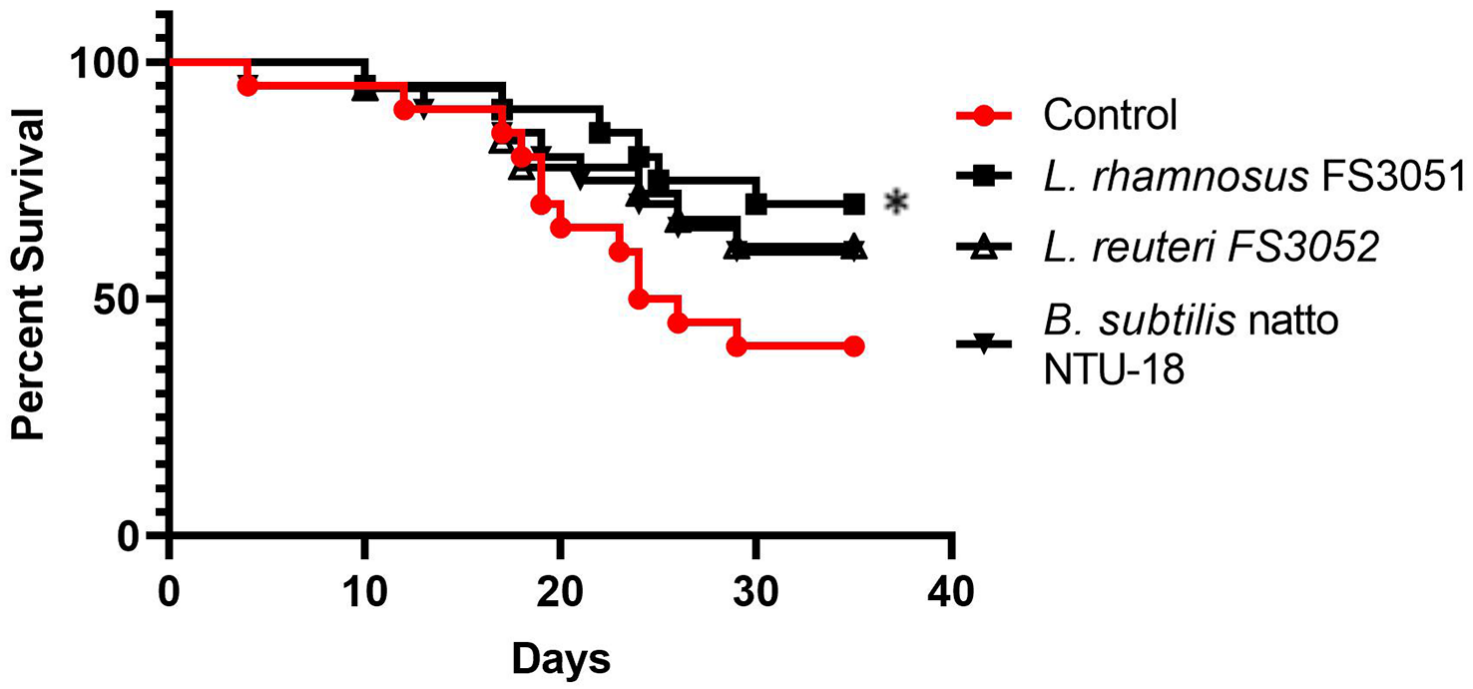
Survival rate of grey mullet fed with different probiotics after *N. seriolae* challenge. * *p* < 0.05. *n* = 30

After 35 days, the survival rate of negative control (fish fed without probiotics) was 35%. The grey mullet fed with *L. rhamnosus* FS3051 had 70% of survival rate in the 35 days post infection, which was significantly higher than the negative control fish. *L. reuteri *FS3052, and *B. subtilis* natto NTU-18 also enhanced the survival rate of grey mullet to 60%, alert non-significant difference (Fig. 2). The *N. seriolae* were detected in the liver of all dead fish.

### The gut microbiota was influenced by *L. rhamnosus* FS3051

**Fig. 3:**
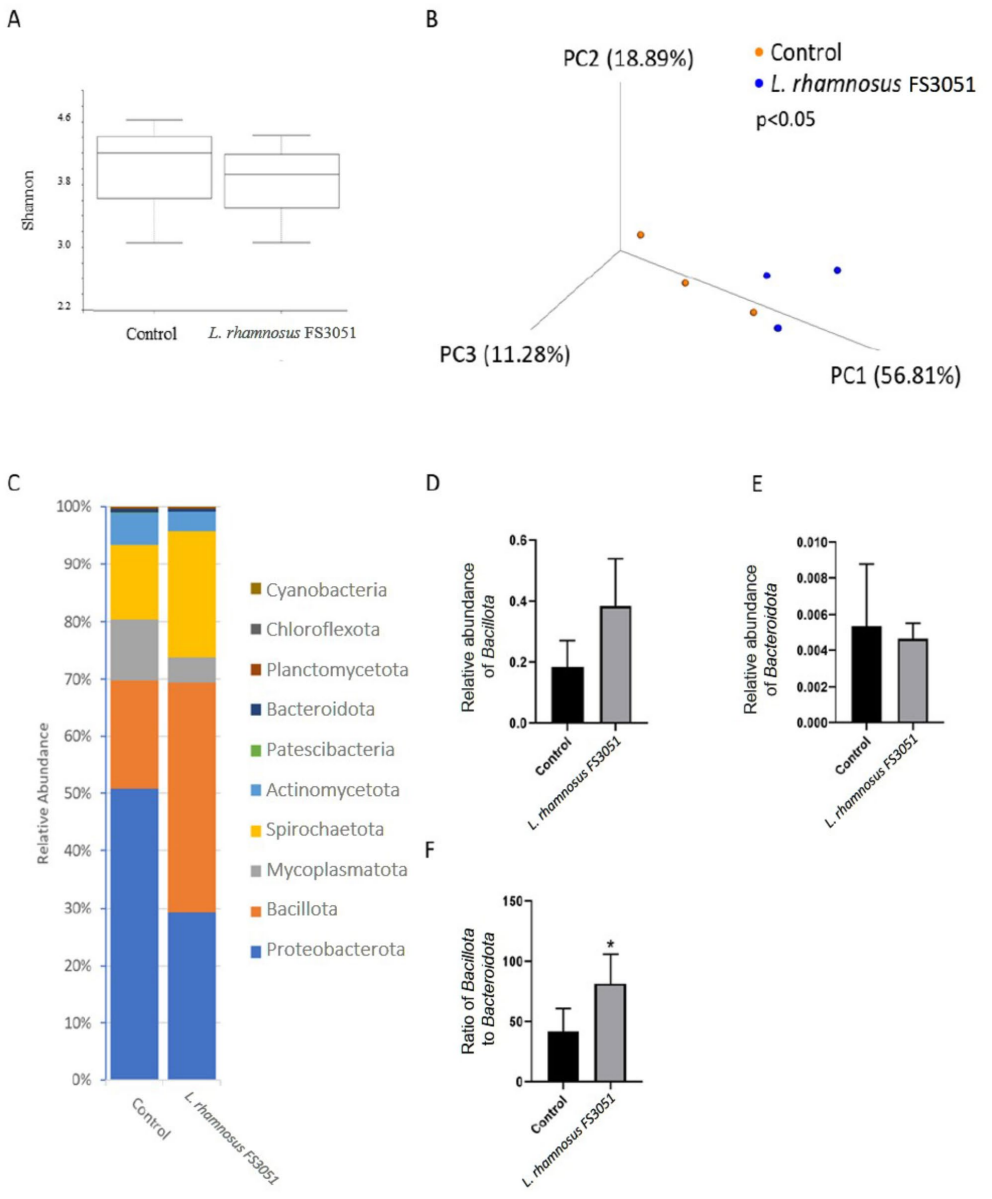
Analysis of gut microbiota. (**A**) Shannon analysis; (**B**) PCoA analysis; (**C**) stacked bar plot of phylum; (**D**) Relative abundance of *Bacillota* (formerly *Firmicutes*); (**E**) Relative abundance of *Bacteroidota* (formerly *Bacteroidetes*); (**F**) Ratio of *Bacillota* to *Bacteroidota*. * *p* < 0.05. *n* = 3

**Fig. 4:**
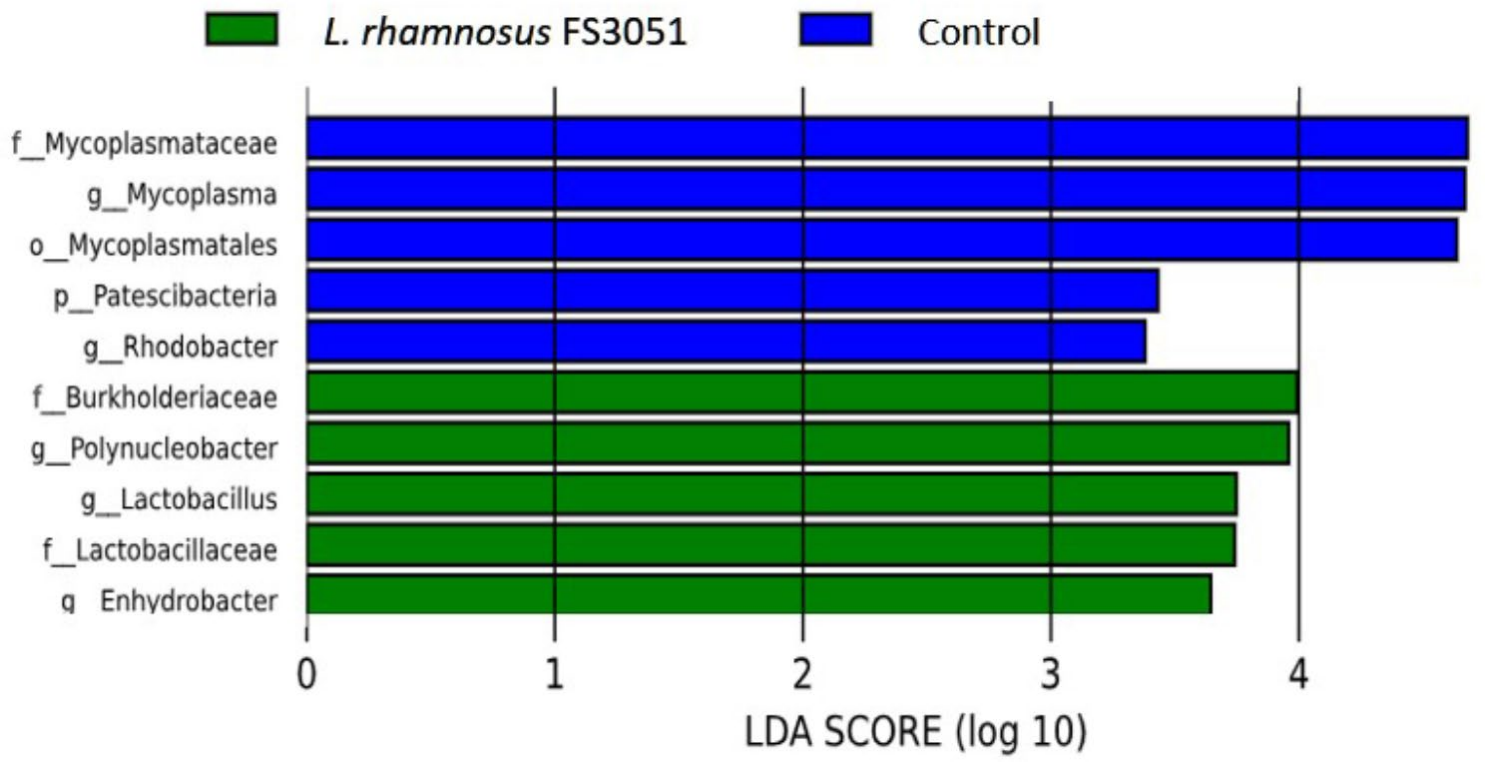
LEfSe analysis of gut microbiota. The log-transformed LDA score of 2 as the threshold. *n* = 3

Because *L. rhamnosus* FS3051 could enhance both growth and resistance against *N. seriolae* in grey mullet, we further analyzed the gut microbiota to evaluate the possible mechanism of *L. rhamnosus* FS3051 in growth and protection against *N. seriolae*. The OTU table from the sequencing analysis is provided in the supplementary file. The result revealed that alpha-diversity was not different between the *L. rhamnosus* FS3051 and control groups (Fig. 3A). The beta-diversity analysis demonstrated that the composition of gut microbiota was altered by *L. rhamnosus* FS3051 (Fig. 3B). The top 10 phyla identified were *Proteobacterota*, *Bacillota* (formerly *Firmicutes*), *Mycoplasmatota*, *Spirochaetota*, *Actinobactetota*, *Patescibacteria*, *Bacteroidota* (formerly *Bacteroidetes*), *Planctomycetota*, *Chloroflexota*, and *Cyanobacteria* (Fig. 3C). Although the relative abundance of *Bacillota* (Fig. 3D) and *Bacteroidota* (Fig. 3E) was not different between the *L. rhamnosus* FS3051 and control groups, the ratio of *Bacillota* to *Bacteroidota* was higher in the *L. rhamnosus* FS3051 than control groups (Fig. 3F). The result of LEfSe indicated that *Polynuceobacter*, *Lactobacillus*, and *Enhydrobacter* were enriched in the genus level in the *L. rhamnosus* FS3051 group, while *Mycoplasma* and *Rhodobacter* were enriched in the control group (Fig. 4).

### Correlation of gut microbiota with immune responses and growth

**Fig. 5:**
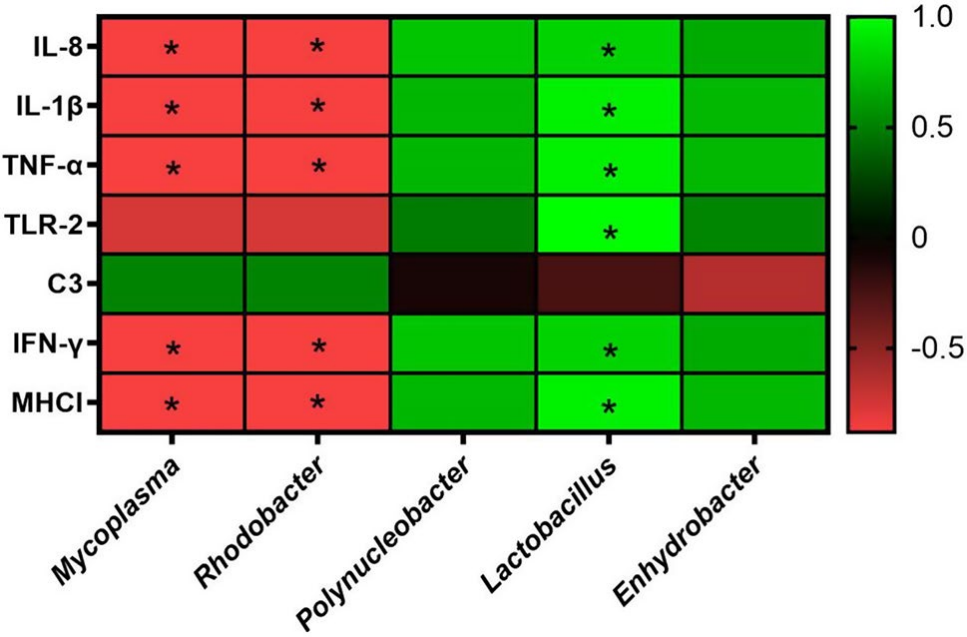
Spearman’scorrelation between the relative abundance of bacterial genera and expression of different immune genes. * *p* < 0.05. *n* = 3

**Fig. 6:**
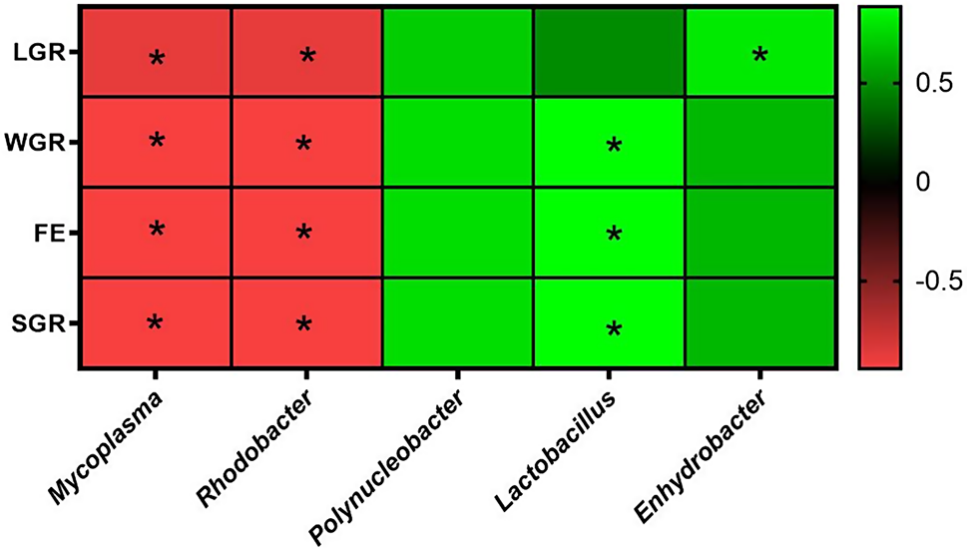
Spearman’scorrelation between the relative abundance of bacterial genera and growth parameters. * *p* < 0.05. *n* = 3

Since the *L. rhamnosus* FS3051 altered the gut microbiota and increased the immune responses, correlations between gut microbiota and immune responses were further analyzed. The results revealed that *Mycoplasma* and *Rhodobacter*, which were diminished in the *L. rhamnosus *FS3051-fed fish, were negatively correlated with IL-8, IL-1β, TNF-α, IFN-γ, and MHCI. Conversely, *Lactobacillus*, which was enriched in the *L. rhamnosus *FS3051-fed fish, was positively correlated with IL-8, IL-1β, TNF-α, TLR-2, IFN-γ, and MHCI (Fig. 5). Growth parameters (WGR, FE, and SGR) were positively correlated with *Lactobacillus* and negatively correlated with *Mycoplasma* and *Rhodobacter*. LGR was positively correlated with *Enhydrobacter* and negatively correlated with *Mycoplasma* and *Rhodobacter* (Fig. 6).

## Discussion

Although probiotics have shown positive effects on growth and disease resistance in various farmed fish, there has been no study focusing on the benefits of probiotics in grey mullet (*Mugil cephalus*) against the major pathogen, *N. seriolae*. This study provides the first evidence that administering the probiotics *L. rhamnosus *FS3051, *L. reuteri *FS3052, and *B. subtilis* natto NTU-18 to grey mullet over 28 days can significantly promote growth and enhance immune responses. The probiotics induced the expression of immune-related genes such as IL-8, IL-1β, TNF-α, TLR-2, C3, IFN-γ, and MHC I. Additionally, these treatments increased the survival rates of grey mullet following infection with *N. seriolae*. Notably, the gut bacterial genus *Lactobacillus*, which was elevated by *L. rhamnosus* FS3051 supplementation, showed a positive correlation with both immune responses and growth. Conversely, the bacterial genera *Rhodobacter* and *Mycoplasma*, which were reduced by the treatment, were negatively correlated with these outcomes. Furthermore, *L. rhamnosus* FS3051 altered the gut microbiota that was correlated with immune responses. These immune responses were involved in the anti-*N. seriolae* functions, including anti-bacteria pro-inflammation and anti-intracellular pathogen [29–32]. Therefore, *L. rhamnosus* FS3051 may protect grey mullet against *N. seriolae* by regulating gut microbiota to induce growth and immune responses.

Probiotics have been suggested to achieve effects on host physiology by improving gut microbiota composition [33]. Indeed, altering gut microbiota composition can influence immunity, pathogen resistance, and growth [33–35]. However, the concept of probiotics has not been extensively addressed in grey mullet. However, similar findings have been reported in other fish studies [36–40]. For example, the dietary probiotic *Pediococcus acidilactici* MA18/5 M can modulate the gut microbiota in rainbow trout and subsequently enhance the production of pro-inflammatory cytokines such as IL-1 and IL-8 [36]. The increased relative abundance of *Lactobacillus* in the gut has been associated with enhanced immune responses [38] and growth [37, 38] in Nile tilapia (*Oreochromis niloticus*) and Eurasian carp (*Cyprinus carpio*). Additionally, *Rhodobacter* has been negatively correlated with IL-1β and TNF-α levels in common carp [39], and the negative correlation showed between *Rhodobacter* and the levels of SGR and WGR in yellow catfish (*Pelteobagrus fulvidraco*) [40]. It was not surprising, as the lipopolysaccharide of *Rhodobacter* has been reported to block the TLR2 pathway, leading to an inhibition of pro-inflammatory responses [41]. Moreover, *Rhodobacter* has been suggested to compete for ecological niches with segmented filamentous bacteria, which are known to enhance the growth of yellow catfish [40]. However, the extract of *Rhodobacter* was indicated to promote proinflammation and growth in red tilapia (*Oreochromis mossambicus × Oreochromis niloticus*) [42]. Therefore, the role of *Rhodobacter* in grey mullet need further investigation. *Mycoplasma* was reported as a normal inhabitant of the gut of fishes with an unclear role in the health of fish [43, 44]. Ou et al. reported the similar results to our study that the higher relative abundance of *Mycoplasma* in the gut microbiota was associated with lower level of TNF-α and IL-8 in tiger puffer (*Takifugu rubripes*) [45]. Moreover, the increase of *Bacillota*/*Bacteroidota* ratio by *L. rhamnosus* FS3051 could be another potential reason for the higher growth rate observed in *L. rhamnosus *FS3051-treated fish compared to control group in the present study, because an increase of *Bacillota*/*Bacteroidota* ratio was linked to enhanced energy harvest and weight gain in hosts [46, 47]. A similar change was observed in fast-growing transgenic common carp (*Cyprinus carpio L.*), where it also suggested as a contributing factor to rapid growth [48]. Taken together, *L. rhamnosus* FS3051 likely achieves its benefits of promoting anti-pathogen immunity and growth in grey mullet by regulating the gut microbiota. However, the specific effects of bacteria in gut microbiota in grey mullet are still unknown, necessitating further study to provide more comprehensive evidence.

Probiotics are commonly suggested to enhance immune stimulation and anti-pathogen resistance in fish, particularly those from the *Lactobacillus* and *Bacillus* genera. *L. rhamnosus* has been successfully used as a feed additive in farmed fish such as *Oncorhynchus mykiss* [49], *Oreochromis niloticus* [50], and *Cyprinus carpio* [51], to induce innate immune responses and to prevent diseases. inducing innate immune responses and preventing diseases. Similarly, *B. subtilis* natto NTU-18 and *L. reuteri* FS3052 have shown comparable effects in Japanese eel (*Anguilla japonica*) and Nile Tilapia (*Oreochromis niloticus*), respectively [17, 52]. In this study, the results demonstrated that *L. rhamnosus *FS3051,* B. subtilis* natto NTU-18, and *L. reuteri* FS3052 promoted innate immunity, as evidenced by the elevated expression of at least three innate immune genes in probiotic-fed fish compared to the control group. TLR2, IL-1β, TNF-α, and IL-8 are pro-inflammatory genes that play crucial roles in combating bacterial pathogens. TLR2, a member of the Toll-like receptor family, has a highly conserved structure involved in detecting Gram-positive bacterial cell walls, and its activation leads to the expression of pro-inflammatory cytokines such as IL-1β, TNF-α, and IL-8 [29–31]. IL-1β and TNF-α were suggested to play a crucial role in reducing the bacterial load of *N. seriolae* in orange-spotted grouper (*Epinephelus coioides*) [20]. Suet al. (2021) suggested that vaccines inducing high levels of these genes provided better protection against the Gram-positive bacterial pathogen *Lactococcus garvieae* in grey mullet [21]. Li et al. also reported that the induction of IL-1β, TNF-α, and IL-8 was associated with high vaccine protection against *N. seriolae* in hybrid snakehead [53]. Moreover, the increase of C3 has been linked to enhanced protection of grey mullet against bacterial infections [21]. Therefore, the probiotics are likely able to enhance survival following *N. seriolae* infection, not only as a dietary supplement but also as an adjuvant to vaccines, due to their ability to induce pro-inflammatory immune responses. Additionally, immune genes related to the response against intracellular pathogens, such as IFN-γ and MHC I, were upregulated in fish fed with probiotics. Since *N. seriolae* can cause intracellular infections [54], and recombinant IFN-γ has been shown to protect Ginbuna (*Carassius auratus langsdorfii*) against *N. seriolae* [32]. Thus, the upregulation of IFN-γ and MHCI likely contributed to the increased survival rate in probiotic-fed fish following *N. seriolae* infection.

There is ongoing debate about whether single or multi-strain probiotics are more effective. Pillinger et al. [16] and Xie et al. [55] suggested that multi-strain probiotics show higher efficacy than single strains because each strain can provide different effects and reduce the emergence of resistance in target pathogens. However, other studies indicated that selecting a probiotic with evidence-based trials of efficacy is more important than the number of strains, as single strains were often equivalent to mixtures [56]. In our study, we examined the effects of three individual probiotics to better understand how each one influences disease resistance in grey mullet against *N. seriolae*. This approach was taken because no previous research has specifically explored the impact of probiotics on this particular disease in grey mullet. The results revealed that *L. rhamnosus* FS3051 had the best effect on resistance of *N. seriolae*, with abilities to directly inhibit *N. seriolae* and stimulate pro-inflammatory and anti-intracellular pathogen immunity. In contrast, *B. subtilis* natto NTU-18 and *L. reuteri* FS3052 provided weaker protection and lacked direct inhibition of *N. seriolae* and anti-intracellular pathogen immunity, respectively. Therefore, the conditions for suppressing *N. seriolae* likely include both direct inhibition of the pathogen and induction of a comprehensive immune response. Additionally, *B. subtilis* natto NTU-18 was the only probiotic among the three strains that enhanced the expression of C3, a component of the complement system that assists the host in combating *N. seriolae*. Therefore, a multi-strain probiotic formula containing both *L. rhamnosus* FS3051 and *B. subtilis* natto NTU-18 may offer better efficacy against *N. seriolae* in grey mullet. However, further studies are necessary to confirm this idea and improve the strategy of probiotic supplementation against *N. seriolae* in grey mullet.

There were some limitations in this study. First, the study investigated only in fry. Thus, the present study might not reflect the phenomenon in stages other than fry. Second, the gut microbiota was analyzed in the control and *L. rhamnosus* FS3051 groups with only three fish per group due to the best effects observed with *L. rhamnosus* FS3051 and a limited budget. Therefore, future studies should investigate the gut microbiota of the other probiotics and consider different growth stages to provide a more comprehensive understanding of the probiotic effects on grey mullet.

In conclusion, this study highlights the remarkable benefits of probiotics, especially *L. rhamnosus *FS3051, in boosting the health and survival of grey mullet. *L. rhamnosus* FS3051 not only significantly improved growth rates (WGR, FE, SGR) but also enhanced immune responses (IL-8, IL-1β, TNF-α, IFN-γ, MHCI), resulting in a 70% survival rate against *N. seriolae*. Additionally, it favorably altered the gut microbiota to achieve the effects on immunity and growing performance. These findings position *L. rhamnosus* FS3051 as a powerful, sustainable alternative to antibiotics in aquaculture, promising enhanced fish health and resilience. Our findings could provide a novel strategy to reduce the risk of *N. seriolae* infection and improve the growth performance of grey mullet.

## Electronic supplementary material

Below is the link to the electronic supplementary material.

Supplementary Material 1

Supplementary Material 2

## Data Availability

Data is provided within supplementary information file.
